# Evaluating the Expression and Prognostic Value of Genes Encoding Microtubule-Associated Proteins in Lung Cancer

**DOI:** 10.3390/ijms232314724

**Published:** 2022-11-25

**Authors:** Natsaranyatron Singharajkomron, Varalee Yodsurang, Suthasinee Seephan, Sakkarin Kungsukool, Supinda Petchjorm, Nara Maneeganjanasing, Warunyu Promboon, Wadsana Dangwilailuck, Varisa Pongrakhananon

**Affiliations:** 1 Department of Pharmacology and Physiology, Faculty of Pharmaceutical Sciences, Chulalongkorn University, Bangkok 10330, Thailand; 2 Preclinical Toxicity and Efficacy, Assessment of Medicines and Chemicals Research Unit, Chulalongkorn University, Bangkok 10330, Thailand; 3 Pharmaceutical Sciences and Technology Graduate Program, Faculty of Pharmaceutical Sciences, Chulalongkorn University, Bangkok 10330, Thailand; 4 Respiratory Medicine Department, Central Chest Institute of Thailand, Muang District, Nonthaburi 11000, Thailand; 5 Division of Anatomical Pathology, Central Chest Institute of Thailand, Muang District, Nonthaburi 11000, Thailand

**Keywords:** diagnosis, gene expression, lung cancer, microtubule-associated proteins, prognosis

## Abstract

Microtubule-associated proteins (MAPs) play essential roles in cancer development. This study aimed to identify transcriptomic biomarkers among MAP genes for the diagnosis and prognosis of lung cancer by analyzing differential gene expressions and correlations with tumor progression. Gene expression data of patients with lung adenocarcinoma (LUAD) and lung squamous cell carcinoma (LUSC) from the Cancer Genome Atlas (TCGA) database were used to identify differentially expressed MAP genes (DEMGs). Their prognostic value was evaluated by Kaplan–Meier and Cox regression analysis. Moreover, the relationships between alterations in lung cancer hallmark genes and the expression levels of DEMGs were investigated. The candidate biomarker genes were validated using three independent datasets from the Gene Expression Omnibus (GEO) database and by quantitative reverse transcription polymerase chain reaction (qRT-PCR) on clinical samples. A total of 88 DEMGs were identified from TCGA data. The 20 that showed the highest differential expression were subjected to association analysis with hallmark genes. Genetic alterations in *TP53, EGFR, PTEN, NTRK1,* and *PIK3CA* correlated with the expression of most of these DEMGs. Of these, six candidates—*NUF2, KIF4A, KIF18B, DLGAP5, NEK2,* and *LRRK2*—were significantly differentially expressed and correlated with the overall survival (OS) of the patients. The mRNA expression profiles of these candidates were consistently verified using three GEO datasets and qRT-PCR on patient lung tissues. The expression levels of *NUF2, KIF4A, KIF18B, DLGAP5, NEK2,* and *LRRK2* can serve as diagnostic biomarkers for LUAD and LUSC. Moreover, the first five can serve as prognostic biomarkers for LUAD, while *LRRK2* can be a prognostic biomarker for LUSC. Our research describes the novel role and potential application of MAP-encoding genes in clinical practice.

## 1. Introduction

Based on histology, most patients suffering from non-small cell lung cancer (NSCLC) are diagnosed with two main subtypes, namely lung adenocarcinoma (LUAD, 40%) and lung squamous cell carcinoma (LUSC, 25%) [1]. The survival rate of lung cancer remains low due to its high metastatic potential and chemotherapeutic resistance, making it the major cause of cancer-related death [2]. Moreover, the specific symptoms of lung cancer are unclear; about 70% of patients are diagnosed only at an advanced stage, which highly correlates with low 5-year survival rates [1,3]. Treatment of lung cancer depends on the type of cancer and diagnosis stage. For the NSCLC, surgical resection combined with chemotherapy or chemoradiation is recommended for the early stage; however, most of the patients undergo disease relapse, recurrence, and metastasis. The guidelines have recommended targeted therapy for the advanced stage treatment, in which molecular biomarker testing is required for appropriate drug selection [4,5]. As a drug target, the genetic alteration status of several genes, such as *EGFR*, *ALK*, *KRAS*, *ROS1*, *BRAF*, *NTRK1/2/3*, *MET*, and *RET*, was indicated as predictive biomarkers, but approximately 30% of patients do not carry these kinds of genetic alterations [4,5,6]. In daily clinical practice, only a few diagnosis biomarkers have been used, including thyroid transcription factor 1 (TTF-1) and p40 for immunohistochemistry (IHC) and cytokeratin 19 fragment (CYFRA 21-1) and carcinoembryonic antigen (CEA) for blood/serum testing. The addition of novel markers is required to improve specificity and sensitivity [7]. Prognostic prediction is important to classify the patient’s risk and decide the treatment strategy. Until now, numerous molecular biomarkers have been reported, but there is still no effective prognostic biomarker for clinical use [7]. Therefore, identifying diagnostic and prognostic biomarkers is urgently needed and may provide precise diagnostic tools and therapeutic targets to improve the clinical outcome.

Microtubule dynamics play an essential role in several cellular processes, including cell division, cell motility, cell morphology maintenance, cell signaling, and intracellular trafficking [8]. Perturbations in microtubule dynamics are tightly associated with cancer cell behaviors [9,10]. Microtubules reorganize to facilitate cancer-related activities, such as mitosis and migration, contributing to tumor growth and metastasis, respectively [9,10,11]. In addition, microtubule instability participates in signaling related to cancer cell survival, cell death, and stress response [9,12]. The expression levels and functional changes in microtubule-associated proteins (MAPs), key regulators of microtubule dynamics, were shown to correlate with the prognosis and clinical outcomes of several cancers [13,14,15]. For example, MAPs, such as tau, MAP2, and MAP4, are biomarkers for responsiveness to microtubule-targeting drugs [16,17,18]. Based on these data, MAPs are crucial for tumor development and aggressiveness. However, their roles and clinical application in lung cancer are still poorly understood.

Several MAPs have reported significantly altered expression in various cancers. However, there are a number of MAPs that differently express in each cancer type; for example, MAP2 upregulation was found in several types of carcinoma and myeloma, especially, and it was indicated as a potential tumor marker among neuronal epithelial tumor subtypes due to its limited expression in neurons. Contrastingly, MAP2 was rarely expressed in metastatic melanoma [19,20]. Upregulation of MAP4 was distinctly found in esophageal squamous cell carcinoma, prostate cancer, and lung adenocarcinoma, but its downregulation was observed in oral squamous cell carcinomas, which was associated with poor differentiation and proliferation [18,19]. Likewise, kinesins were elevated in cancers, and *KIFAP3* and *KIF3A* were highly expressed in breast cancer, while these genes were unchanged in lung cancer as to whether there are several *KIFs* reported from our results [19,20]. These data have pointed out the cancer-type specific expression of MAPs and, therefore, we extensively performed transcriptomic analysis to identify their expressions relevant to lung cancer prognosis.

Bioinformatic analyses of gene expression and genetic alteration profiles obtained from international public databases, such as the TCGA program and Gene Expression Omnibus (GEO), have revealed potential biomarkers and drug targets in various cancers [21,22,23]. The differential expression of several genes has been associated with lung cancers and their specific subtypes, including LUAD and LUSC [24,25,26,27,28]. However, specific targets still need to be identified and validated, and their functions and prognostic relevance need to be examined.

The aim of this study was to investigate the differential expression of MAP genes in LUAD and LUSC by analyzing TCGA data. The association between DEMGs and lung cancer hallmark genes was evaluated, and the prognostic and predictive value of DEMGs was assessed. In addition, the potential biomarker genes were validated using three GEO datasets and quantitative reverse transcription polymerase chain reaction (qRT-PCR) tests on patient lung specimens.

## 2. Results

### 2.1. Identification of DEMGs between Tumor and Normal Lung Tissues

**Figure 1:**
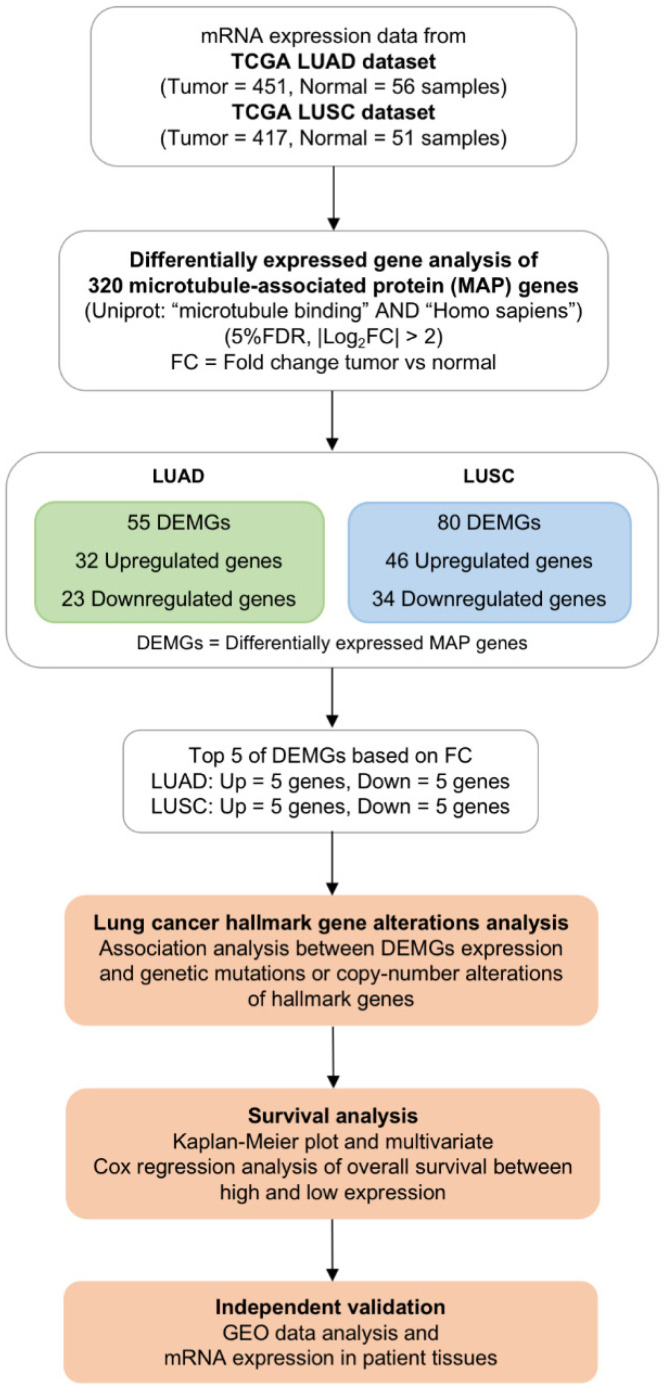
Schematic workflow for screening microtubule-associated protein (MAP)-encoding gene biomarkers.

**Figure 2:**
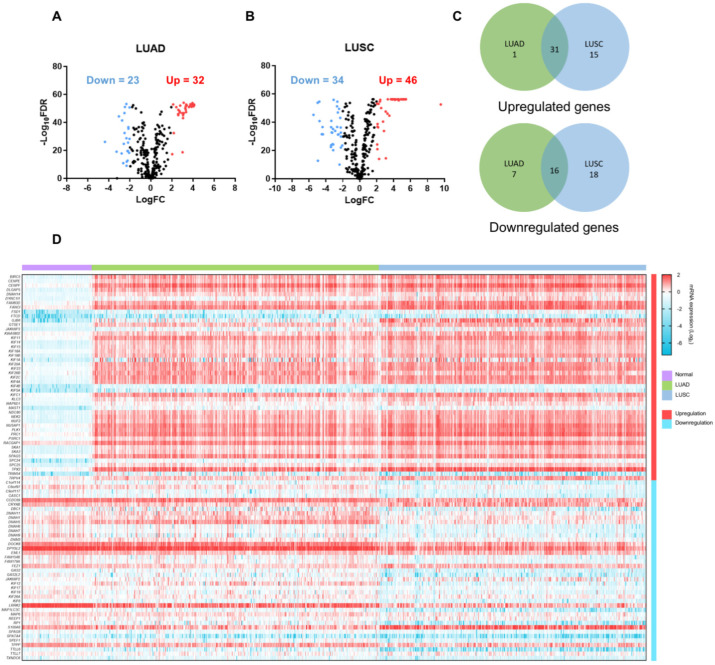
Differentially expressed MAP genes (DEMGs). Volcano plots depict the DEMGs in (**A**) lung adenocarcinoma (LUAD) and (**B**) lung squamous cell carcinoma (LUSC). Red indicates upregulated genes with log fold change (log_2_FC) > 2; blue indicates downregulated genes with log_2_FC < −2, false discovery rate (FDR) < 0.05. (**C**) Venn diagrams represent the intersection of upregulated or downregulated genes between LUAD and LUSC. (**D**) Heatmap represents the mRNA expression (log_2_) of the DEMGs in normal, LUAD, and LUSC samples.

To screen for transcriptomic biomarkers, 320 MAP genes were identified from the UniProt database using the search terms “microtubule binding” AND “Homo sapiens” [29]. The workflow of this study is shown in Figure 1. Gene expression levels retrieved from the TCGA database were compared between tumor and normal lung tissues. The clinical characteristics of patients from the TCGA database are shown in Table 1. Sex, age, and race of patients were comparable between the tumor (*n* = 868) and normal (*n* = 107) groups. There were 451 LUAD and 417 LUSC tumor tissues. Volcano plots were used to display the significance of and fold change in DEMGs (FDR < 0.05, |log_2_FC| > 2) identified in each subtype (Figure 2A,B). Differential expression analysis of TCGA data demonstrated 32 upregulated and 23 downregulated genes in LUAD, and 46 upregulated and 34 downregulated genes in LUSC. Among DEMGs, 31 upregulated and 16 downregulated genes overlapped between the 2 subtypes (Figure 2C). The heatmap represents the expression level of each DEMG in normal, LUAD, and LUSC samples (Figure 2D, Supplementary Figure S1). The top five most upregulated genes in the LUAD dataset included *NUF2, KIF4A, KIF18B, NEK2,* and *DLGAP5,* while those in the LUSC dataset included *NUF2, KIF4A, KIF18B, GJB6,* and *BIRC5.* The top five most downregulated genes in the LUAD dataset were *DNAH9, SPATA4, LRRK2, FAM154B,* and *REEP1*, while those in the LUSC dataset were *RP1, MAP1LC3C, DNAH11, LRRK2,* and *TTLL6*. These 20 DEMGs were selected for subsequent analyses. Here, *NUF2, KIF4A,* and *KIF18B* were commonly upregulated, and *LRRK2* was commonly downregulated in both lung cancer subtypes.

### 2.2. Association between DEMG Expressions and Lung Cancer Hallmark Gene Alterations

**Figure 3:**
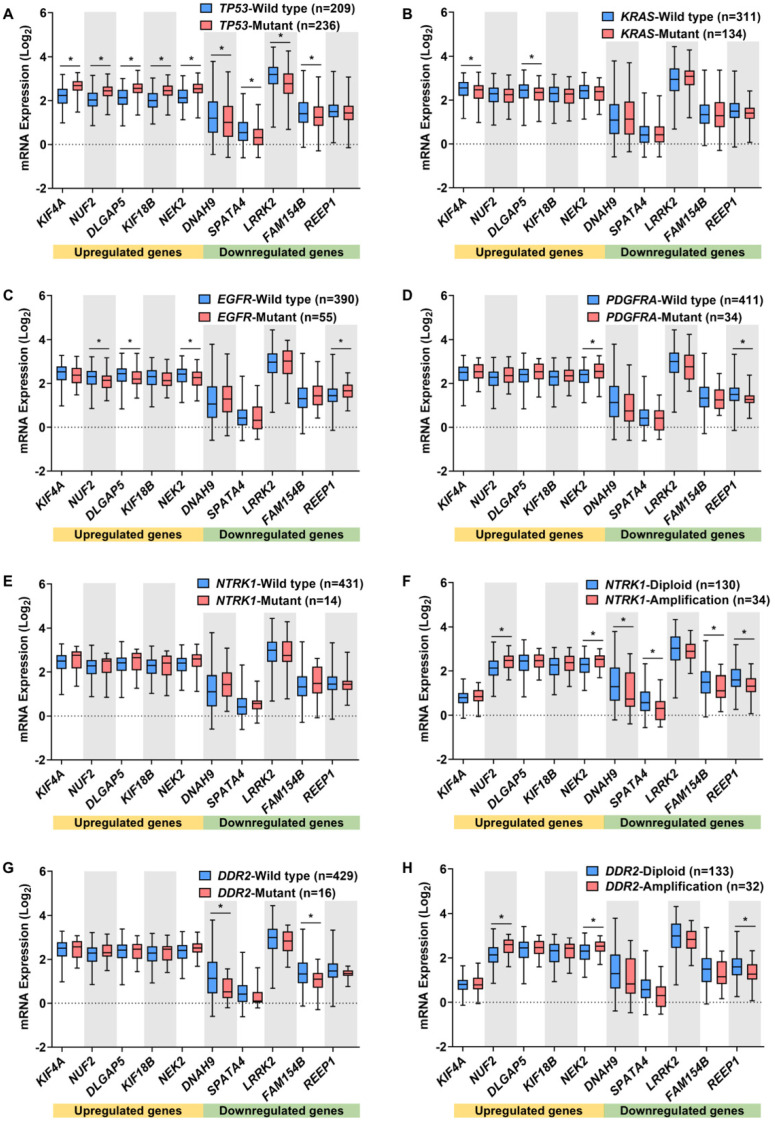
Relationships between the top five DEMG expressions in LUAD and its lung cancer hallmark gene alterations. Box plots demonstrate the association between DEMG expressions and mutation of *TP53* (**A**), *KRAS* (**B**), *EGFR* (**C**), *PDGFRA* (**D**), *NTRK1* (**E**), and *DDR2* (**G**), and amplification of *NTRK1* (**F**), and *DDR2* (**H**). * *p* < 0.05 vs. wild-type or diploid group.

**Figure 4:**
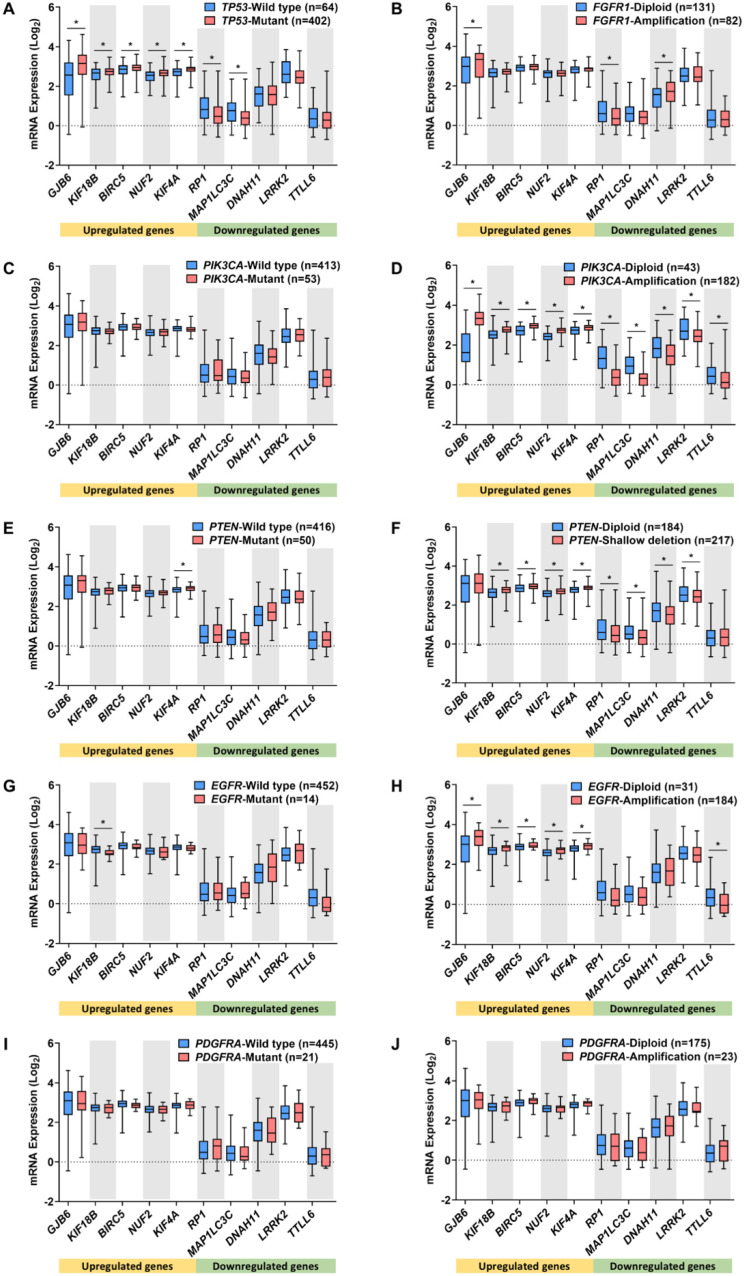
Relationships between the top five DEMG expressions in LUSC and its lung cancer hallmark gene alterations. Box plots demonstrate the association between DEMG expressions and mutation of *TP53* (**A**), *PIK3CA* (**C**), *PTEN* (**E**), *EGFR* (**G**), and *PDGFRA* (**I**), amplification of *FGFR1* (**B**), *PIK3CA* (**D**), *EGFR* (**H**), and *PDGFRA* (**J**), and shallow deletion of *PTEN* (**F**). * *p* < 0.05 vs. wild-type or diploid group.

The regulatory roles of DEMGs in oncogenesis can be discerned by evaluating the association between their expression and the genetic alterations in hallmark genes. The hallmark genes included oncogenes and tumor suppressor genes, whose mutation, amplification, or deletion was strongly associated with the pathology and aggressiveness of lung cancer [1,30,31,32,33,34,35]. The alteration frequencies of hallmark genes in LUAD and LUSC tumor samples were confirmed by cBioPortal [36] and the top five most altered genes were included in this study, as shown in Supplementary Table S1. The mRNA expression levels of the 20 DEMGs were investigated in different alteration profiles of the hallmark genes (Figure 3 and Figure 4).

In LUAD, the mutation of *TP53*, the most frequently altered gene, was significantly related to the expression levels of nine DEMGs (Figure 3A). Compared with *TP53* wild-type, tumors with *TP53* mutations showed significantly higher levels of the upregulated genes *KIF4A, NUF2, DLGAP5, KIF18B,* and *NEK2,* and lower levels of the downregulated genes *DNAH9, SPATA4, LRRK2,* and *FAM154B*, potentially implicating these genes in oncogenesis. Furthermore, *KRAS* mutation status was associated with decreased levels of *KIF4A* and *DLGAP5* (Figure 3B), while patients with an *EGFR* mutation exhibited a decrease in *NUF2, DLGAP5,* and *NEK2* expression and an increase in *REEP1* expression (Figure 3C). Additionally, *NEK2* and *REEP1* expressions were associated with *PDGFRA* mutation (Figure 3D), *NUF2, NEK2, DNAH9, SPATA4, FAM154B,* and *REEP1* expression with *NTRK1* amplification (Figure 3F), *DNAH9* and *FAM154B* expression with *DDR2* mutations (Figure 3G), and *NUF2, NEK2,* and *REEP1* expression with *DDR2* amplification (Figure 3H). The *NTRK1* mutation was not significantly related to the expression of DEMGs (Figure 3E).

In the LUSC cohort, *TP53* mutations were associated with expression changes in the following seven DEMGs: *GJB6, KIF18B, BIRC5, NUF2,* and *KIF4A* were upregulated, while *RP1* and *MAP1L3C3* were downregulated (Figure 4A). Compared with *FGFR1* diploid patients, those with *FGFR1* amplification displayed significantly altered levels of *GJB6, RP1,* and *DNAH11* (Figure 4B). Remarkably, while *PIK3CA* mutations were not associated with fluctuating levels of DEMGs, *PIK3CA* amplification was significantly related to the levels of all DEMGs, as follows: the upregulated DEMGs showed an increase, while the downregulated ones showed a decrease (Figure 4C,D). Shallow deletions and mutations are commonly found in *PTEN* alterations. These *PTEN* shallow deletions were associated with an increase in the expressions of *KIF18B, BIRC5, NUF2,* and *KIF4A*, and a decrease in the expressions of *RP1, MAP1L3C3, DNAH11,* and *LRRK2* (Figure 4F). The *PTEN* mutations were related to the high expression of *KIF4A* (Figure 4E). Moreover, only the low level of *KIF18B* expression was associated with *EGFR* mutations (Figure 4G), while the high expressions of *GJB6, KIF18B, BIRC5, NUF2,* and *KIF4A,* and the low expression of *TTLL6*, were found in patients with *EGFR* amplification (Figure 4H). The expression of DEMGs did not significantly alter with *PDGFRA* mutation and amplification in LUSC tissues (Figure 4I,J).

### 2.3. Prognostic Value of DEMGs

**Figure 5:**
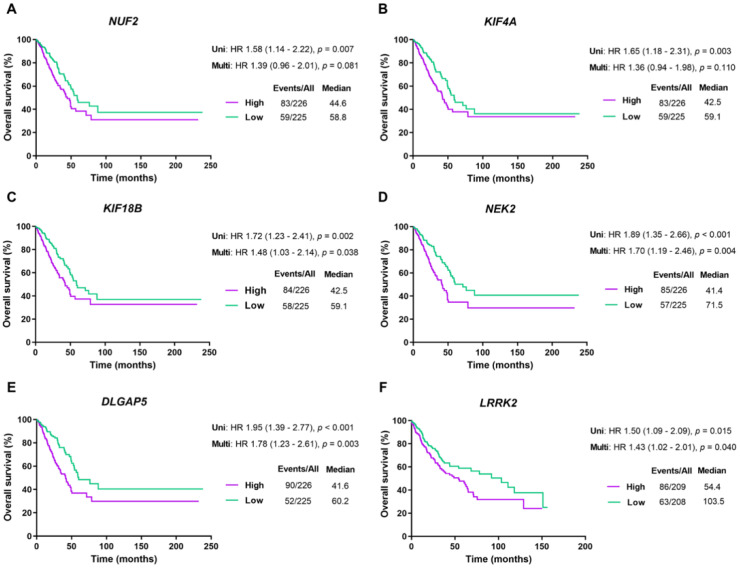
Survival analysis of the top five DEMGs in LUAD and LUSC patients. Kaplan–Meier curves present the overall survival rate of *NUF2* (**A**), *KIF4A* (**B**), *KIF18B* (**C**), *NEK2* (**D**), and *DLGAP5* (**E**) in LUAD, and *LRRK2* (**F**) in LUSC. Abbreviations are as follows: Uni, univariate analysis; Multi, multivariate analysis; HR, hazard ratio; Events/All, No. of events/No. of patients; Median (months).

The prognostic value of the 20 DEMGs was evaluated via the Kaplan–Meier method [37]. Based on the median expression level of each gene, patients from the TCGA dataset were stratified into either low- or high-expression groups. Statistical analysis using a logrank test suggested that the high expressions of *NUF2, KIF4A, KIF18B, NEK2,* and *DLGAP5* in LUAD (logrank *p* = 0.007, 0.003, 0.002, <0.001, <0.001, respectively) and *LRRK2* in LUSC (logrank *p* = 0.015) were significantly related to worse OS for the patients (Figure 5).

Furthermore, univariate and multivariate Cox regression analyses were applied to assess the independent predictive value of each gene for the survival outcomes of lung cancer patients, including OS, disease-specific survival (DSS), and progression-free survival (PFS). Since *TP53* mutation status was related to the expression of most DEMGs and significantly correlated with survival outcomes in both LUAD and LUSC patients (Supplementary Tables S2 and S3), it was included as a covariate in the multivariate analysis of the identified DEMGs.

In LUAD patients, the clinical stage and the expression of *NUF2, NEK2,* and *DLGAP5* were significantly correlated with all types of survival outcomes, while the expression of *KIF4A* and *KIF18B* and *TP53* mutation status were significantly correlated with OS and DSS in univariate analyses. In the multivariate analysis, the expression of *NEK2* and *DLGAP5* was related to all types of survival outcomes, that of *NUF2* was associated with DSS and PFS, and that of *KIF18B* was correlated with OS (Supplementary Table S2).

In the LUSC dataset, the clinical stage significantly correlated with all types of survival outcomes, *TP53* mutation status correlated with OS and DSS in univariate analyses, and *LRRK2* expression was associated with OS in both univariate and multivariate analyses (Supplementary Table S3). Taken together, the high expression of six genes was remarkably associated with poor survival outcomes and independently correlated with the prognosis of lung cancer patients.

### 2.4. Gene Set Enrichment Analysis (GSEA) of DEMGs

To further evaluate the associated pathway of the DEMGs, we perform the GSEA using gene expression data from the TCGA, and hallmark gene sets from the Molecular Signatures Database (MsigDB). We compared the datasets for high- and low-expression of each gene. Considering the most significantly enriched signaling pathways based on normalized enrichment score (NES), high expression of *NUF2, KIF4A, KIF18B, DLGAP5*, and *NEK2* and low expression of *LRRK2* were mostly associated with G2/M checkpoint, E2F targets, MTORC1 signaling, and MYC targets. In addition, *KIF4A, KIF18B*, and *DLGAP5* upregulation was associated with an increment in the mitotic spindle regulation, and elevation of *NEK2* was linked to an increase in unfolded protein response (Supplementary Figure S2). As the roles of the G2/M checkpoint, E2F targets, MTORC1 signaling, MYC targets, and mitotic spindle regulation were mainly implicated in the cell cycle progression, this finding supported the significant role of candidate genes in tumor growth, and suggests that their function might participate in these pathways.

### 2.5. Validation of the Six Candidate Biomarkers Using Patient Samples and GEO Databases

**Figure 6:**
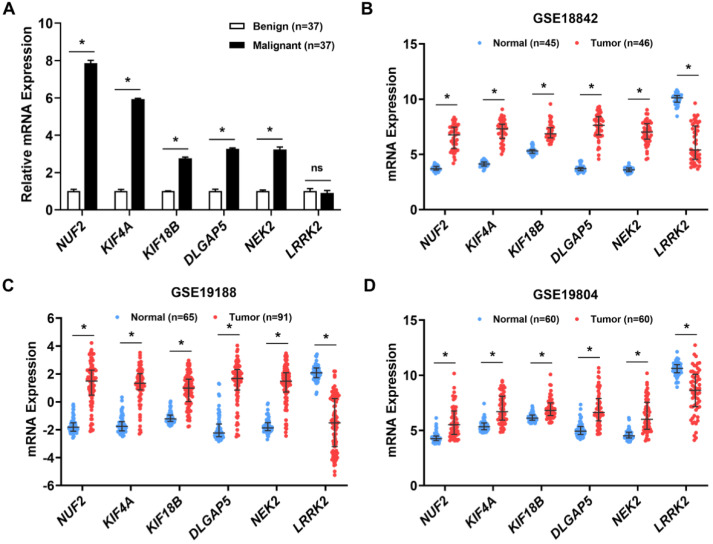
Validation of the candidate DEMG expressions. Clinical validation in benign (*n* = 37) and malignant (*n* = 37) lung tissues was analyzed by quantitative reverse transcription polymerase chain reaction (**A**). * *p* < 0.05 vs. benign tissue, ns = not significant. The DEMG expressions were examined in the following three Gene Expression Omnibus datasets: GSE18842 (**B**), GSE19188 (**C**), and GSE19804 (**D**), compared between normal and tumor tissues. * *p* < 0.05 vs. normal tissue.

The six candidate biomarkers, associated with hallmark gene alterations and poor survival outcomes of patients, were verified for their differential expression using patient lung tissues and GEO databases. These candidates included five upregulated genes—*NUF2, KIF4A, KIF18B, NEK2,* and *DLGAP5*—and one downregulated gene—*LRRK2*—in the tumor tissues of TCGA databases. The expression levels of these biomarkers were measured by qRT-PCR in patient lung biopsy tissues that had pathologically been classified as benign (*n* = 37) or malignant (*n* = 37). The baseline clinical characteristics of patients in the two groups were not significantly different, except for age, which was lower in the benign group (*p*-value = 0.030) (Table 2). The mRNA levels of *NUF2, KIF4A, KIF18B, DLGAP5,* and *NEK2* were significantly more than two-fold higher in malignant tissues than in benign tissues. However, *LRRK2* expression levels did not differ significantly between the two groups (Figure 6A). Although age was significantly different between the two groups (Table 2), the expressions of these candidate genes were age-independent (Supplementary Figure S3). To further validate these biomarkers, the expression of the six candidate genes was compared between normal and tumor lung tissues from three independent GEO datasets, namely GSE18842, GSE19188, and GSE19804. Consistently, *NUF2, KIF4A, KIF18B, DLGAP5,* and *NEK2* were highly overexpressed, whereas *LRRK2* was significantly downregulated in the tumor tissues of all datasets (Figure 6B–D). These results corroborate the evidence indicating the involvement of these six candidate genes in lung cancer pathogenesis.

The relevance of DEMGs in lung cancer was further confirmed by the in vitro experiments. We first determined the proliferation profile of several lung cancer cell lines. Then, the cells were classified into a high and low proliferation based on their relative cell proliferation values. The mRNA expression of candidate genes was compared between the high proliferative lung cancer cell lines (A549 and H460 cells) and the low proliferative lung cancer cell lines (H292 and H23 cells). The results demonstrated that *NUF2, KIF4A, KIF18B, DLGAP5, and NEK2* were higher expressed, while *LRRK2* levels were lower in the cells that have a greater proliferative activity (Supplementary Figure S4). These data, at least, provided the oncogenic and tumor suppressive effect of the upregulated and downregulated candidate genes in lung cancer, respectively, supporting our findings from the bioinformatic and clinical sample analyses.

## 3. Discussion

Lung cancers display aggressive characteristics with metastasis and chemotherapeutic resistance [38,39]. Most patients are only diagnosed at an advanced stage due to non-specific symptoms, leading to the high progression and high death rate of lung cancer [1,3]. Identifying novel biomarkers for earlier diagnosis or prognosis could improve the clinical outcomes of patients. Multiple studies have reported that MAPs contribute to several cancer-related processes, including tumor growth, metastasis, and chemoresistance [13,14,15,20]. Several mitosis-associated genes, including *AURKA, DLGAP5, TPX2, KIF11,* and *CKAP5*, were overexpressed in tumor tissues, and associated with cell proliferation and poor OS [40]. Despite much evidence implicating MAPs in the pathogenesis of lung cancer, over 300 MAPs are still unexplored. In the present study, the transcriptomics of 320 MAPs were investigated, and the highly differentially expressed ones that were associated with alterations in hallmark genes and lung cancer prognosis were characterized. For the first time, we propose *NUF2, KIF4A, KIF18B, DLGAP5, NEK2*, and *LRRK2* as biomarkers for lung cancer progression.

The NUF2 biomarker is a component of the nuclear division cycle 80 complex, which serves to segregate chromosomes during cell division [41]. Pan-cancer analysis in 31 distinct tumor types has revealed the overexpression of *NUF2* in 23 cancer types. In contrast, it showed no significant alteration in kidney, prostate, and thyroid cancers, while its downregulation was found in leukemia and testicular cancers [42]. Silencing of NUF2 suppresses an in vitro cell proliferation and inhibits tumor growth in pancreatic and liver cancers [43,44]. We demonstrated the consistent upregulation of *NUF2* in LUAD and LUSC datasets, patient tumor tissues, and GEO datasets, supporting the muti-omics analysis that its protein level was upregulated in clinical tissues [45,46]. Furthermore, *NUF2* knockdown was shown to induce cell death and cell invasion in lung cancer cell lines [47], indicating the potential role of *NUF2* in lung cancer progression. Furthermore, functional enrichment analysis revealed roles of *NUF2* in the cell cycle and p53 signaling, mutations which resulted in the overexpression of *NUF2* [45,46]. Similarly, we found that *TP53* mutation was strongly related to the upregulation of *NUF2* in both LUAD and LUSC, suggesting that *TP53* negatively regulates *NUF2*.

The gene encoding EGFR plays an oncogenic role in lung cancer, and its mutation and amplification have been commonly recorded [31,48]. The expressions of *EGFR* and *NUF2* were found to be positively correlated [46]. We further demonstrated that *EGFR* mutation was associated with decreasing expression of *NUF2* in LUAD and, interestingly, *EGFR* amplification, mostly identified in LUSC, was highly related to the increased expression of *NUF2* in this subtype. These data highlight the distinct influence of *EGFR* in regulating *NUF2* in lung cancer subtypes, but their interaction at the molecular level remains to be elucidated.

Mutations in *NTRK1* and *DDR2*, which encode tyrosine kinase receptors, have been reported in lung cancer [32]. The rearrangement of *NTRK1* constitutively activates the receptor and its downstream signaling, which mediates tumor growth [33]. When DDR2 binds to extracellular collagen, it triggers SHP-2, SRC, and mitogen-activated protein kinase signaling; hence, *DDR2* mutations induce cancer cell proliferation, differentiation, and metastasis [49,50]. In the present study, amplifications of *NTRK1* and *DDR2* were associated with elevated expression of *NUF2* only in the LUAD subtype. However, the regulatory roles of *NTRK1* and *DDR2* amplification on lung cancer cell behaviors have not yet been characterized.

We found that *PIK3CA* amplification was associated with the upregulation of *NUF2* in LUSC. Here, *PIK3CA* encodes phosphatidylinositol-3-kinase, which plays an important role in activating the Akt signaling pathway, the regulator of several cancer-related activities [51]. Mutation or amplification of *PIK3CA* has been reported and associated with lung cancer progression [52]. Consistent with our study, a positive correlation between *PIK3CA* and *NUF2* expression has been reported [46].

In addition, we showed that shallow deletion of *PTEN*, a well-known tumor suppressor, was tightly correlated with increased expression of *NUF2* in LUSC. Indeed, PTEN, a phosphatase enzyme, suppresses cancer cell growth through its downstream transcriptional activity [53]. Our data suggest that PTEN negatively regulates *NUF2* expression, but the exact regulatory mechanism needs to be further elucidated. Overall, our findings provide a more detailed picture of the regulation of *NUF2* in lung cancer and indicate that its expression can independently predict the DSS and PFS outcomes in LUAD patients.

Furthermore, KIF4A and KIF18B, members of the kinesin superfamily, execute essential functions in microtubule trafficking [54]. Overexpression of *KIF4A* and *KIF18B* was observed in various cancers [55,56]. The previous study revealed that KIF4A promotes cell proliferation by inducing p21-mediated cell cycle progression in colorectal cancer [57]. In contrast, *KIF4A* was downregulated in gastric cancer, and its elevation inhibits the proliferation of human gastric carcinoma cells [58]. Additionally, KIF18B participates in the Wnt/beta-catenin signaling pathway that induces cell proliferation, migration, and invasion in cervical and breast cancers [59,60]. In cases of lung cancer, KIF4A was reported to promote cell proliferation and migration and inhibit apoptosis in LUAD cell lines, whereas KIF18B was suggested to promote LUAD cell proliferation, migration, and invasion via the Rac1/Akt/mammalian target of rapamycin (mTOR) signaling pathway [61,62]. Roles of *KIF4A* and *KIF18B* have been substantially reported in LUAD [61,62,63], but the present study provides new evidence suggesting their potential roles in LUSC. The overexpression and clinical relevance of *KIF4A* and *KIF18B* have also been previously established through cross-validation between the public databases and clinical samples [61,62,63].

We discovered that *TP53* mutation correlated with increased *KIF4A* and *KIF18B* expression in LUAD and LUSC. Supporting this finding, elevated levels of KIF4A were encountered in *TP53*-mutant compared with wild-type lung cancer cell lines, indicating the negative regulation of this candidate gene by *TP53* [64]. Tumor suppressor p53, a transcription factor, participates in microtubule organization by regulating the expression of tubulins and MAPs [9]. Therefore, p53 might govern *KIF4A* and *KIF18B* expression through its transcriptional activity.

Furthermore, *KRAS*, which encodes a GTPase downstream of the tyrosine kinase receptor, is an essential mediator for cancer cell growth, differentiation, and apoptosis [65]. In this study, *KRAS* mutation was related to decreased expression of *KIF4A* in LUAD. In contrast, an analysis of cBioPortal data revealed that *KIF4A* was upregulated in *KRAS*-mutant pancreatic ductal adenocarcinoma patient tissues [66]. It is possible that *KRAS* might regulate *KIF4A* in a cell type-specific manner, and this particular regulation in the context of lung cancer requires further elucidation.

Additionally, *EGFR* amplification was found to be correlated with increased expression of *KIF4A* and *KIF18B*, while *EGFR* mutation was related to the decreased expression of *KIF18B* in LUSC. Since EGFR initiates signal transduction through a network of downstream pathways that activate transcription of target genes [67], such as signal transducer and activator of transcription 3 (STAT3), extracellular signal-regulated kinase (ERK), and mTOR, it might indirectly regulate *KIF4A* and *KIF18B* transcription via these pathways. In addition, the multivariate analysis suggested that *KIF18B* was an independent predictor of OS in LUAD. Taken together, these findings demonstrate the impact of these MAP genes in the oncogenic regulation of lung cancer hallmark genes.

Furthermore, DLGAP5, a microtubule stabilizer, plays a crucial role in the formation of tubulin polymers [40]. Cohort and bioinformatic studies have revealed high expression of *DLGAP5* and its association with poor OS in numerous cancers [40,68]. Thus, *DLGAP5* knockdown could attenuate cell growth and induce apoptosis via cyclin-dependent kinase 1/cyclin D1/Bcl-2 signaling in ovarian cancer cells [69]. Consistent with these findings, we found that *DLGAP5* was upregulated in LUAD, positively associated with *TP53* mutation, and negatively associated with *KRAS* and *EGFR* mutation, indicating the tumor oncogenic activity of *DLGAP5* in relation to the lung cancer hallmark genes. Moreover, it showed the potential to predict OS, DSS, and PFS in LUAD, making it a promising prognostic biomarker.

Likewise, the serine/threonine protein kinase *NEK2* was upregulated in multiple cancers [70]. Targeting NEK2 inhibits tumorigenesis through the Wnt1/beta-catenin signaling pathway in cervical cancer [71]. Furthermore, overexpression of NEK2 in triple-negative breast cancer cells promotes cell migration and invasion [72]. Additionally, *NEK2* has also been found to be overexpressed and possess significant prognostic value in lung cancer [68,73]. It is known that NEK2 plays an important role in stabilizing microtubules during mitotic processes. Elevated levels of NEK2 induce cell proliferation and chromosome instability in NSCLC cells [74]. We provided consistent evidence for the upregulation and prognostic value of *NEK2* in LUAD and found that *TP53* and *PDGFRA* mutations were correlated with its elevated expression. The *TP53* genetic lesion was previously found to be correlated with the amplification/overexpression of *NEK2* in multiple myeloma, suggesting *NEK2* as a promising target in *TP53*-mutant myeloma [75]. Interestingly, *EGFR* mutation was reported to induce *NEK2* expression via the ERK signaling pathway, promoting in vitro NSCLC cell proliferation, and this NEK2 upregulation was found to impair the sensitivity of EGFR-targeting drugs [76]. We also found increased levels of *NEK2* in clinical specimens with *EGFR* mutation. We further indicated that amplification of the hallmark genes *NTRK1* and *DDR2* may be related to the upregulation of *NEK2*, but further clarification is needed to accurately define their association.

Furthermore, *LRRK2*, a well-known regulator in Parkinson’s disease, was recently reported to be downregulated and associated with lung cancer progression [77,78], consistent with our findings. This LRRK2 was shown to participate in host immune responses involving the recruitment of macrophages in the tumor microenvironment, and its overexpression was reported in kidney and thyroid cancers [79]. The depletion of LRRK2 inhibited cell proliferation and migration and induced thyroid cancer cell apoptosis by inhibiting the c-Jun N-terminal kinase signaling pathway [80]. However, its role in lung cancer was largely unknown. Here, we have reported its involvement in lung cancer type for the first time and identified the relationship between *TP53* mutation and decreased expression of *LRRK2* in LUAD. In contrast, *PIK3CA* amplification and *PTEN* shallow deletion were correlated with the upregulation of *LRRK2*. However, we found no difference in the expression level of *LRRK2* in patient tissues from our cohort. Therefore, the differential expression of *LRRK2* in tumor versus normal tissues must be validated with a larger sample size to accurately discern its regulatory role in lung cancer.

These identified molecules also exhibit potential drug targets; however, there is no inhibitor approved for all candidate genes. Even some inhibitors are in the processes of in vitro, in vivo, and clinical investigation. Indeed, NUF2 and DLGAP5 inhibitors have not been reported. This is similar to KIF4A and KIF18B, although several KIF-inhibitors are undergoing clinical trials. For example, Ispinesib, a KIF11 inhibitor, was evaluated in a phase I clinical trial in breast cancer, although the specific inhibitors for KIF4A and KIF18B are still limited [81]. While NEK2 inhibitors have been widely established, and the efficacy was tested in both in vitro and in vivo in several types of cancer, including multiple myeloma, leukemia, gastric, colorectal, glioma, breast, and liver cancers, their effect on lung cancer requires further investigation [82]. As a key target for Parkinson’s disease, inhibitors for LRRK2 have been developed [83], but the anticancer effect of this inhibitor has not yet been evaluated. In cancer research, a recent study has demonstrated the role of LRRK2 only in thyroid cancer [80]. Likewise, our study has highlighted the significance of LRRK2 in lung cancer; however, the effect of LRRK2 inhibitors in the cancer context requires further clarification. Taken together, our study revealed the significant alterations of these genes in lung cancer in a subtype-specific manner. Further in vitro and in vivo investigations could strengthen the impact of these molecules in lung cancer cell biology and provide an advantage for clinical application.

## 4. Materials and Methods

### 4.1. Gene Expression Datasets

The mRNA expression data and clinicopathological data of LUAD and LUSC patients from the TCGA database were obtained from cBioPortal (https://www.cbioportal.org/ accessed on 4 August 2021) [36]. The LUAD dataset included 507 samples (451 tumors and 56 normal tissues), while the LUSC dataset included 468 samples (417 tumors and 51 normal tissues). Samples lacking survival and clinical data were excluded from the study. The clinical characteristics of patients are shown in Table 1.

### 4.2. Differential Expression Analysis

The differential expression of MAP genes between tumor and normal samples was analyzed using the R program version 2022.02.0+443 [84]. The false discovery rate (FDR) was calculated by Benjamini–Hochberg adjustment of the *p*-value. Fold change (FC) was used to quantify the difference in mRNA expression levels. The MAP genes with FDR < 0.05 and |log_2_FC| > 2 were determined as DEMGs. The DEMGs were presented in a volcano plot, Venn diagram, and heatmap, which were all generated using GraphPad Prism 9. From each dataset, the top five DEMGs exhibiting the highest fold changes were selected for subsequent analyses.

### 4.3. Lung Cancer Hallmark Gene Alteration Analysis

Genetic alteration profiles of lung cancer hallmark genes in LUAD and LUSC, including mutations and copy-number variations from the TCGA database, were obtained from cBioPortal (https://www.cbioportal.org/, accessed on 4 August 2021) [36]. The top five altered hallmark genes from each dataset, shown in Supplementary Table S1, were included in the analysis. Gene expression levels of DEMGs were compared between patients with wild-type and mutant genes or among patients with diploid, amplified, or deleted hallmark genes.

### 4.4. Survival Analysis

Kaplan–Meier plots of the OS of patients, categorized by high or low mRNA expression determined by the median expression of DEMGs [27,85], were generated using GraphPad Prism 9. The *p*-value was calculated by the logrank test [37]. Patients in the high expression group were characterized by their expression levels being above the median, while those in the low expression group showed expression levels below the median. Univariate and multivariate analyses were performed by the Cox’s proportional hazards regression model using a stepwise selection of variables. Clinicopathological variables, including age, sex, clinical stage, and *TP53* mutation status, were selected for the analyses. A *p*-value less than 0.05 was considered statistically significant.

### 4.5. Patients and Tissue Samples

A total of 74 lung cancer patients from the Central Chest Institute of Thailand were enrolled in this study after obtaining written informed consent from them. All experiments were approved by the Central Research Ethics Committee of the Central Chest Institute of Thailand (approval number 086/2563) and were performed in accordance with the Helsinki Declaration of 1975. Lung biopsy samples were collected and defined as benign or malignant by the pathologist. The clinical information of patients is summarized in Table 2. Fresh lung tissues were frozen in RNA stabilizing solution (Invitrogen, Carlsbad, CA, USA) and stored at −20 °C for subsequent biochemical assays.

### 4.6. qRT-PCR

Total RNA was extracted from the patient’s lung tissues using GENEzol^TM^ reagent (Geneaid, New Taipei City, Taiwan) and reverse-transcribed to cDNA using the iScript cDNA Synthesis Kit (Bio-Rad Laboratories, CA, USA), following the manufacturer’s instructions. Then, qRT-PCR was performed using SensiFAST™ SYBR Green Supermix (Meridian Bioscience, OH, USA). The expression levels were normalized to the internal control, glyceraldehyde-3-phosphate dehydrogenase, and relative expressions were calculated by the 2^−∆∆Ct^ method. Primers used in this study are shown in Supplementary Table S4.

### 4.7. GEO Data Validation

To validate the associations of candidate biomarkers, three lung cancer datasets (i.e., GSE18842, GSE19188, GSE19804), containing gene expression data on tumor and normal tissues, were used for an independent analysis. The gene expression profile from the microarray and corresponding clinical information were obtained from GEO (https://www.ncbi.nlm.nih.gov/gds, accessed on 7 September 2022) [22]. The GSE18842 included dataset 46 tumors and 45 adjacent normal lung tissues. The GSE19188 dataset included 91 tumors and 65 adjacent normal lung tissues. The GSE19804 dataset included 60 tumors and 60 adjacent normal lung tissues.

### 4.8. Statistical Analysis

Statistical analysis was performed using GraphPad Prism 9. Student’s *t*-test and Mann–Whitney U test were used for comparisons between two groups of continuous variables, as appropriate. The chi-square test was applied for comparisons between two groups of categorical variables. A *p*-value less than 0.05 was considered statistically significant.

## 5. Conclusions

The present study identified and validated *NUF2, KIF4A, KIF18B, DLGAP5, NEK2,* and *LRRK2* as promising diagnostic biomarkers for lung cancer. The increased expressions of *NUF2, KIF4A, KIF18B, DLGAP5,* and *NEK2* were prognostic biomarkers in LUAD, and the reduced expression of *LRRK2* was a prognostic biomarker in LUSC. Their gene expression levels were strongly related to alterations in lung cancer hallmark genes, including *TP53, KRAS, EGFR, PDGFRA, NTRK1, DDR2, PIK3CA,* and *PTEN*. Further in vitro and in vivo investigations of the molecular mechanisms underlying these associations might support their clinical application as biomarkers for lung cancer.

## Figures and Tables

**Table 1:** Clinical characteristics of the Cancer Genome Atlas dataset.

Characteristic	Tumor (*n* = 868)	Normal (*n* = 107)	*p*-Value *
Sex―no. (%)			
Female	351 (40.44)	46 (42.99)	0.612
Male	517 (59.56)	61 (57.01)	
Age―years			
Median (Min, Max)	67 (38, 88)	67 (42, 86)	0.330
Overall Survival―no. (%)			
Alive	576 (66.36)		
Deceased	292 (33.64)		
Race―no. (%)			
White	613 (87.20)	93 (92.08)	0.360
Black or African American	73 (10.38)	8 (7.92)	
Asian	16 (2.28)	0 (0)	
American Indian or Alaskan Native	1 (0.14)	0 (0)	
Cancer subtype―no. (%)			
LUAD	451 (51.96)		
LUSC	417 (48.04)		
Stage―no. (%)			
I	457 (52.65)		
II	247 (28.46)		
III	134 (15.44)		
IV	30 (3.46)		

* Calculated based on Mann–Whitney U test and chi-square test. Here, LUAD is lung adenocarcinoma; LUSC is lung squamous cell carcinoma.

**Table 2:** Clinical characteristics of patients for clinical validation.

Characteristic	Malignancy (*n* = 37)	Benign (*n* = 37)	*p*-Value *
Sex―no. (%)			
Female	17 (45.95)	19 (51.35)	0.816
Male	20 (54.05)	18 (48.65)	
Age―years			
Median (Min, Max)	65 (42, 87)	63 (17, 81)	0.030
Smoking―no. (%)			
Yes	18 (48.65)	15 (40.54)	0.640
No	19 (51.35)	22 (59.46)	
Carcinogen exposure―no. (%)			
Yes	7 (18.92)	5 (13.51)	0.754
No	30 (81.08)	32 (74.42)	
Family history―no. (%)			
Yes	8 (21.62)	2 (5.41)	0.085
No	29 (78.38)	35 (94.60)	
Stage―no. (%)			
I	4 (10.81)		
II	9 (24.32)		
III	11 (29.73)		
IV	13 (35.14)		

* Calculated based on a Mann–Whitney U test and chi-square test.

## Data Availability

All data supporting the findings of this study are available within the article and its Supplementary Materials.

## Supplementary Materials

The supporting information can be downloaded at https://www.mdpi.com/article/10.3390/ijms232314724/s1.
