# Supplementary material for: Evaluating the Expression and Prognostic Value of Genes Encoding Microtubule-Associated Proteins in Lung Cancer
Source: Int J Mol Sci. 2022 Nov 25;23(23):14724. doi: 10.3390/ijms232314724 (PMC9738182; doi:10.3390/ijms232314724)
Supplement: Supplementary file 1 [file ijms-23-14724-s001.zip › ijms-2021174-supplementary.pdf]

Supplementary information for

Evaluating the Expression and Prognostic Value of Genes Encoding Microtubule-Associated Proteins in Lung Cancer

Natsaranyatron Singharajkomron [1], Varalee Yodsurang [1,2], Suthasinee Seephan [3], Sakkarin Kungsukool [4], Supinda Petchjorm [5], Nara Maneeganjanasing [1], Warunyu Promboon [1], Wadsana Dangwilailuck [1] and Varisa Pongrakhananon [1,2,*]

[1] Department of Pharmacology and Physiology, Faculty of Pharmaceutical Sciences, Chulalongkorn University, Bangkok 10330, Thailand

[2] Preclinical Toxicity and Efficacy, Assessment of Medicines and Chemicals Research Unit, Chulalongkorn University, Bangkok 10330, Thailand

[3] Pharmaceutical Sciences and Technology Graduate Program, Faculty of Pharmaceutical Sciences, Chulalongkorn University, Bangkok 10330, Thailand

[4] Respiratory Medicine Department, Central Chest Institute of Thailand, Muang District, Nonthaburi 11000, Thailand

[5] Division of Anatomical Pathology, Central Chest Institute of Thailand, Muang District, Nonthaburi 11000, Thailand

***Corresponding author**

Varisa Pongrakhananon

Tel.: +662-218-8325; Fax: +662-218-8340

Email: Varisa.p@pharm.chula.ac.th

Supplementary Materials and Methods

1. *Gene set enrichment analysis (GSEA)*

GSEA was performed using 4.3.2 software. Gene expression data of LUAD and LUSC datasets from the TCGA database were applied with 1000 gene-set permutations using the gene-ranking metric T-test with the collection "h.all.v2022.1.Hs.symbols (Hallmarks)" gene sets obtained from the Molecular Signatures Database (MsigDB) (https://www.gsea-msigdb.org/gsea/msigdb/human/collections.jsp#H). Patients were categorized into high- and low-expression groups based on the median expression of each gene.

2. *Cell culture*

NSCLC cell lines H460 (HTB-177), H292 (CRL-1848), H23 (CRL-5800), and A549 (CCL-185) cell lines were obtained from American Type Culture Collection (ATCC; Manassas, VA, USA). H460, H292, and H23 cells were cultured in Roswell Park Memorial Institute (RPMI) 1640 medium. A549 cells were cultured in Dulbecco's Modified Eagle Medium (DMEM). All cell culture mediums were supplemented with 10% fetal bovine serum (FBS), 2mM L-glutamine, 100 unit/mL penicillin, and 100 ug/mL streptomycin. All media and supplements were purchased from Invitrogen (Carlsbad, CA, USA). Cells were maintained at 37°C in a humidified atmosphere of 5% CO_2.

3. *Cell proliferation assay*

Cell proliferation of lung cancer cell lines were assessed by MTT assay. Cells were seeded onto 96-well plates at the density of 3 x 10^3 cells/well. After 72 h, 10 μL of MTT (5 mg/mL) was then added to each well. After incubation for 4 h, 100 μL of DMSO was added to dissolve the formazan, and the optical density was measured using a microplate reader at 570 nm. The cell proliferation was calculated as a relative value to the initial

time point (0 h). Cells were classified into high (> 2-fold) and low proliferative (≤ 2-fold) cells.

Supplementary Figures and Tables

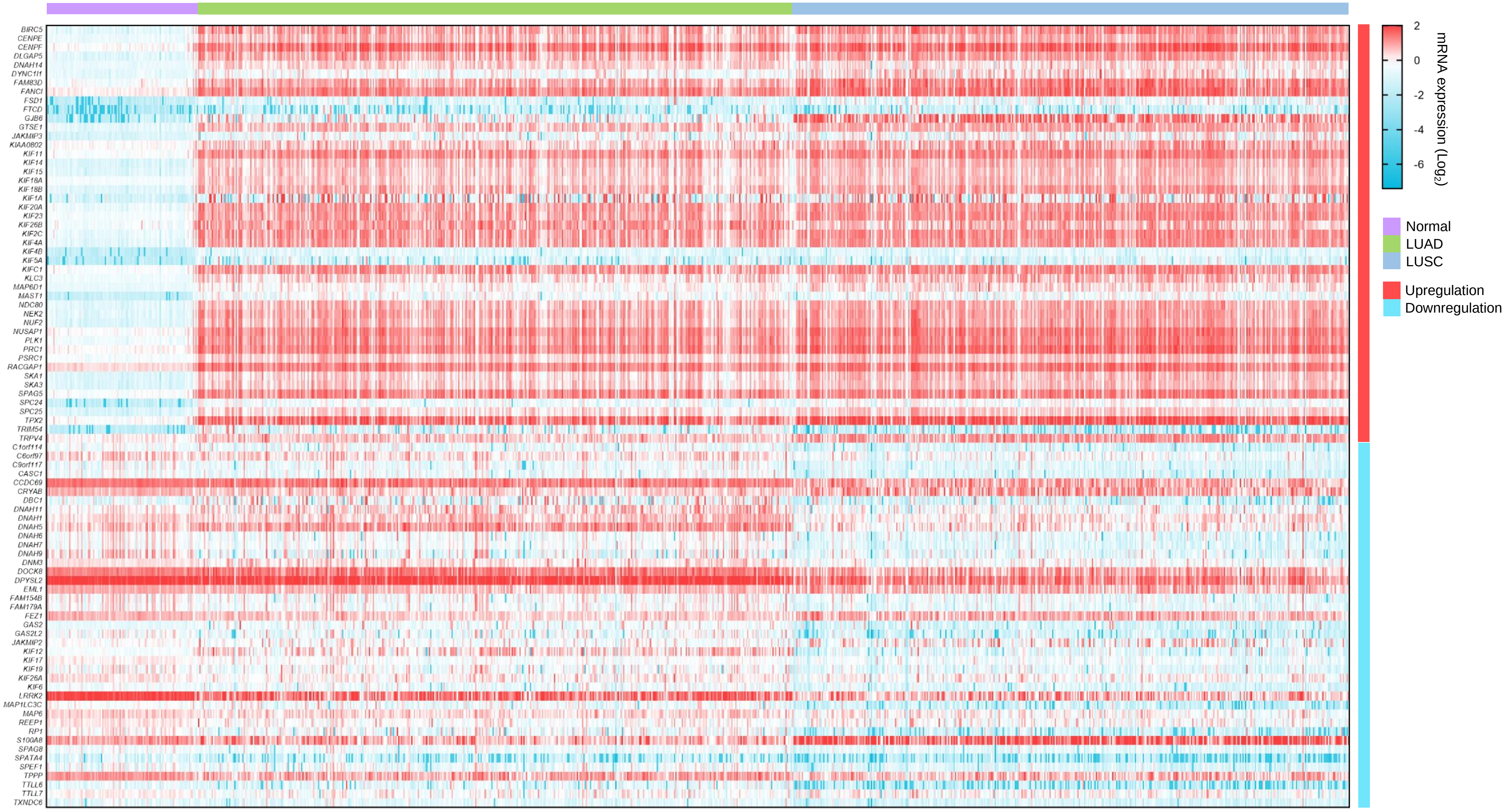

Supplementary Figure S1 Heatmap represents the mRNA expression (log2) of the DEMGs in normal, LUAD, and LUSC samples.

Supplementary Table S1 Genetic alteration profile of lung cancer hallmark genes and related DEMGs in LUAD and LUSC.

Top 5 altered gene*	% Alteration*	Major alteration*	Reported alteration**	Related DEMGs
**LUAD**				
TP53	52	Mutation	Mutation[a]	*KIF4A, NUF2, DLGAP5, KIF18B, NEK2, DNAH9, SPATA4, LRRK2, FAM154B*
KRAS	32	Mutation	Mutation[b,c,d]	*KIF4A, DLGAP5*
EGFR	15	Mutation	Mutation[b,c,d]	*NUF2, DLGAP5, NEK2, REEP1*
NTRK1	9	Amplification	Rearrangement[e]	*NUF2, NEK2, DNAH9, SPATA4, FAM154B, REEP1* (Amplification)
PDGFRA	9	Mutation	Mutation[d]	*NEK2, REEP1*
DDR2	9	Amplification	Mutation[b,c]	*NUF2, NEK2, REEP1* (Amplification), *DNAH9*, *FAM154B* (Mutation)
**LUSC**				
TP53	83	Mutation	Mutation[a]	*GJB6, KIF18B, BIRC5, NUF2, KIF4A, RP1, MAP1LC3C*
PIK3CA	44	Amplification	Mutation[b,c,d,f]	*GJB6, KIF18B, BIRC5, NUF2, KIF4A, RP1, MAP1LC3C, DNAH11, LRRK2, TTLL6* (Amplification)
PTEN	20	Mutation, Shallow deletion	Mutation[b,c,d], Loss[g]	*KIF4A* (Mutation), *KIF18B, BIRC5, NUF2, KIF4A, RP1, MAP1LC3C, DNAH11, LRRK2* (Shallow deletion)
FGFR1	19	Amplification	Amplification[b,f]	*GJB6, RP1, DNAH11*
EGFR	9	Amplification	Mutation[b,c,d]	*GJB6, KIF18B, BIRC5, NUF2, KIF4A, TTLL6* (Amplification), *KIF18B* (Mutation)
PDGFRA	9	Amplification	Mutation[d]	-

*Based on TCGA data (n=451 for LUAD and n=417 for LUSC tumor tissues) obtained from cBioPortal (https://www.cbioportal.org/)

**Based on published literature [a][24], [b][1], [c][25], [d][26], [e][27], [f][28], [g][29]

Supplementary Table S2 Univariate and multivariate Cox regression analyses of the survival outcomes in LUAD.

Variable	Overall survival		Disease-specific survival		Progression-free survival	
	HR (95% CI)	*P* value	HR (95% CI)	*P* value	HR (95% CI)	*P* value
**Univariate Analysis**						
Age	1.00 (0.98-1.02)	0.892	0.99 (0.97-1.01)	0.175	1.00 (0.98-1.01)	0.514
Sex						
Male	1(reference)		1(reference)		1(reference)	
Female	0.90 (0.64-1.25)	0.508	0.99 (0.68-1.46)	0.972	0.90 (0.68-1.21)	0.492
Stage						
I-II	1(reference)		1(reference)		1(reference)	
III-IV	2.37 (1.66-3.34)	**<0.001**	2.38 (1.57-3.53)	**<0.001**	1.57 (1.12-2.17)	**0.007**
Expression (Low vs. High)						
NUF2	1.58 (1.14-2.22)	**0.007**	1.93 (1.31-2.89)	**0.001**	1.47 (1.10-1.97)	**0.009**
KIF4A	1.65 (1.18-2.31)	**0.003**	1.66 (1.13-2.45)	**0.010**	1.32 (0.99-1.77)	0.059
KIF18B	1.72 (1.23-2.41)	**0.002**	1.66 (1.14-2.46)	**0.010**	1.32 (0.99-1.77)	0.058
NEK2	1.89 (1.35-2.66)	**<0.001**	1.98 (1.35-2.96)	**0.001**	1.46 (1.09-1.95)	**0.011**
DLGAP5	1.95 (1.39-2.77)	**<0.001**	2.11 (1.42-3.17)	**<0.001**	1.54 (1.15-2.07)	**0.004**
TP53 (WT vs. MT)	1.53 (1.09-2.16)	**0.014**	1.68 (1.14-2.51)	**0.010**	1.24 (0.93-1.66)	0.150
**Multivariate Analysis**						
Stage						
I-II	1(reference)		1(reference)		1(reference)	
III-IV	2.24 (1.56-3.18)	**<0.001**	2.16 (1.41-3.23)	**<0.001**	1.53 (1.09-2.11)	**0.012**
NUF2 (Low vs. High)	1.39 (0.96-2.01)	0.081	1.73 (1.13-2.69)	**0.014**	1.44 (1.08-1.93)	**0.015**
TP53 (WT vs. MT)	1.26 (0.87-1.82)	0.220	1.28 (0.84-1.97)	0.262		
Stage						
I-II	1(reference)		1(reference)			
III-IV	2.18 (1.51-3.10)	**<0.001**	2.12 (1.38-3.18)	**<0.001**		
KIF4A (Low vs. High)	1.36 (0.94-1.98)	0.110	1.34 (0.87-2.08)	0.188		
TP53 (WT vs. MT)	1.26 (0.87-1.83)	0.226	1.39 (0.91-2.16)	0.132		
Stage						
I-II	1(reference)		1(reference)			
III-IV	2.18 (1.51-3.10)	**<0.001**	2.13 (1.39-3.19)	**<0.001**		
KIF18B (Low vs. High)	1.48 (1.03-2.14)	**0.038**	1.40 (0.92-2.15)	0.125		
TP53 (WT vs. MT)	1.24 (0.86-1.79)	0.252	1.39 (0.91-2.14)	0.129		
Stage						
I-II	1(reference)		1(reference)		1(reference)	
III-IV	2.21 (1.53-3.13)	**<0.001**	2.14 (1.40-3.21)	**<0.001**	1.52 (1.08-2.11)	**0.013**
NEK2 (Low vs. High)	1.70 (1.19-2.46)	**0.004**	1.78 (1.17-2.74)	**0.007**	1.42 (1.06-1.90)	**0.018**
TP53 (WT vs. MT)	1.21 (0.85-1.73)	0.304	1.31 (0.87-2.00)	0.207		
Stage						
I-II	1(reference)		1(reference)		1(reference)	
III-IV	2.14 (1.49-3.04)	**<0.001**	2.07 (1.35-3.10)	**<0.001**	1.49 (1.05-2.06)	**0.020**
DLGAP5 (Low vs. High)	1.78 (1.23-2.61)	**0.003**	1.94 (1.26-3.05)	**0.003**	1.48 (1.11-1.99)	**0.009**
TP53 (WT vs. MT)	1.16 (0.81-1.67)	0.429	1.24 (0.81-1.90)	0.325		

HR, hazard ratio; CI, confidence interval; WT, wild type; MT, mutant.

Supplementary Table S3 Univariate and multivariate Cox regression analyses of the survival outcomes in LUSC.

**Variable**	**Overall survival**		**Disease-specific survival**		**Progression-free survival**	
	**HR (95% CI)**	***P* value**	**HR (95% CI)**	***P* value**	**HR (95% CI)**	***P* value**
**Univariate Analysis**						
Age	1.01 (0.99-1.03)	0.182	0.99 (0.97-1.02)	0.608	0.99 (0.97-1.01)	0.534
Sex						
Male	1(reference)		1(reference)		1(reference)	
Female	0.68 (0.45-1.00)	0.059	0.74 (0.43-1.23)	0.271	0.84 (0.55-1.26)	0.422
Stage						
I-II	1(reference)		1(reference)		1(reference)	
III-IV	1.55 (1.03-2.26)	**0.030**	2.59 (1.59-4.11)	**<0.001**	2.23 (1.46-3.31)	**<0.001**
Expression (Low vs. High)						
LRRK2	1.50 (1.09-2.09)	**0.015**	1.35 (0.87-2.10)	0.189	1.40 (0.98-2.01)	0.070
TP53 (WT vs. MT)	0.51 (0.34-0.81)	**0.003**	0.42 (0.25-0.77)	**0.003**	0.61 (0.38-1.04)	0.052
**Multivariate Analysis**						
Stage						
I-II	1(reference)					
III-IV	1.70 (1.13-2.50)	**0.009**				
LRRK2 (Low vs. High)	1.43 (1.02-2.01)	**0.040**				
TP53 (WT vs. MT)	0.53 (0.35-0.85)	**0.005**				

HR, hazard ratio; CI, confidence interval; WT, wild type; MT, mutant.

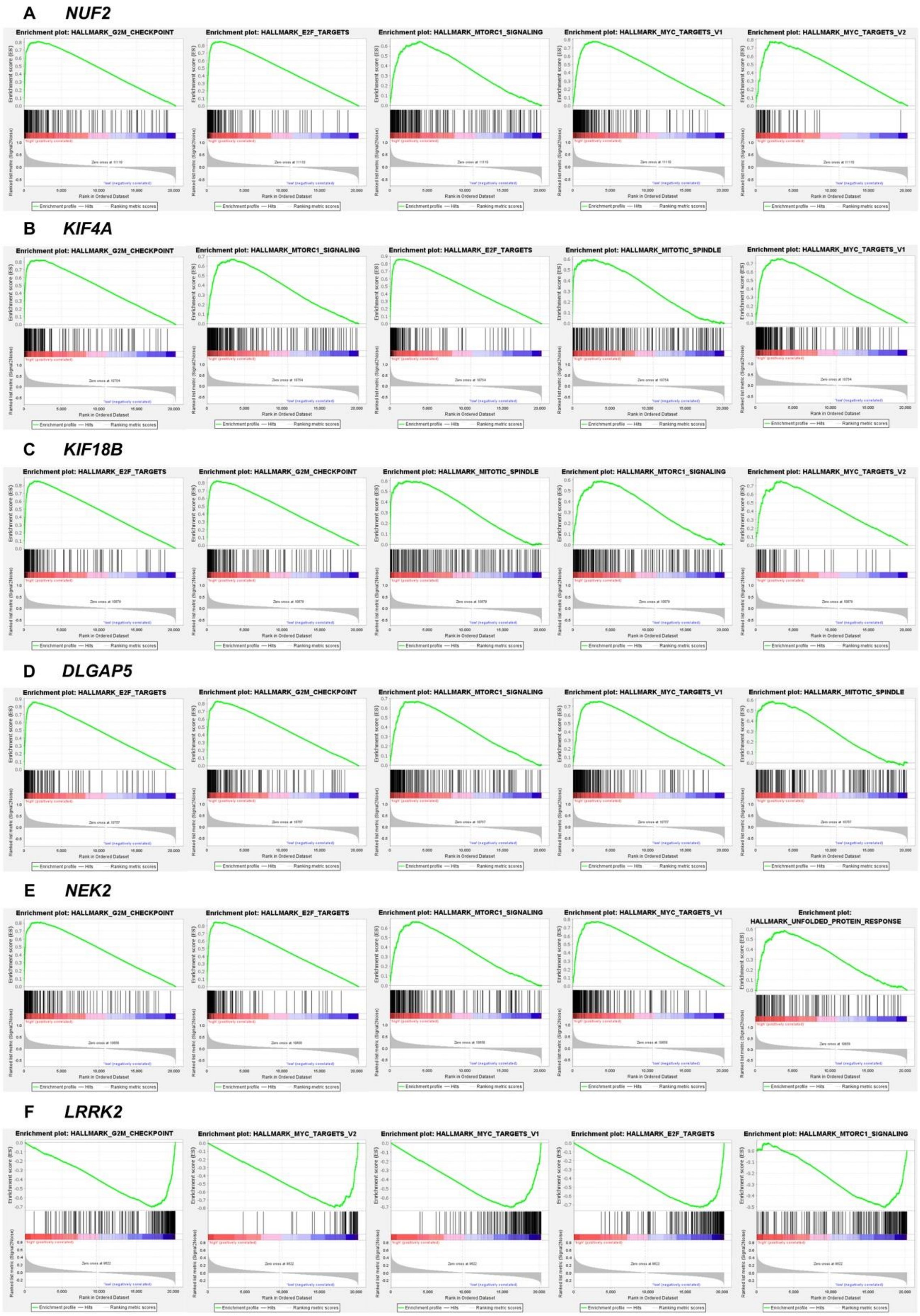

Supplementary Figure S2 Gene set enrichment analysis (GSEA) of candidate genes. Enrichment plots of NUF2 (A), KIF4A (B), KIF18B (C), DLGAP5 (D), NEK2 (E), and LRRK2 (F).

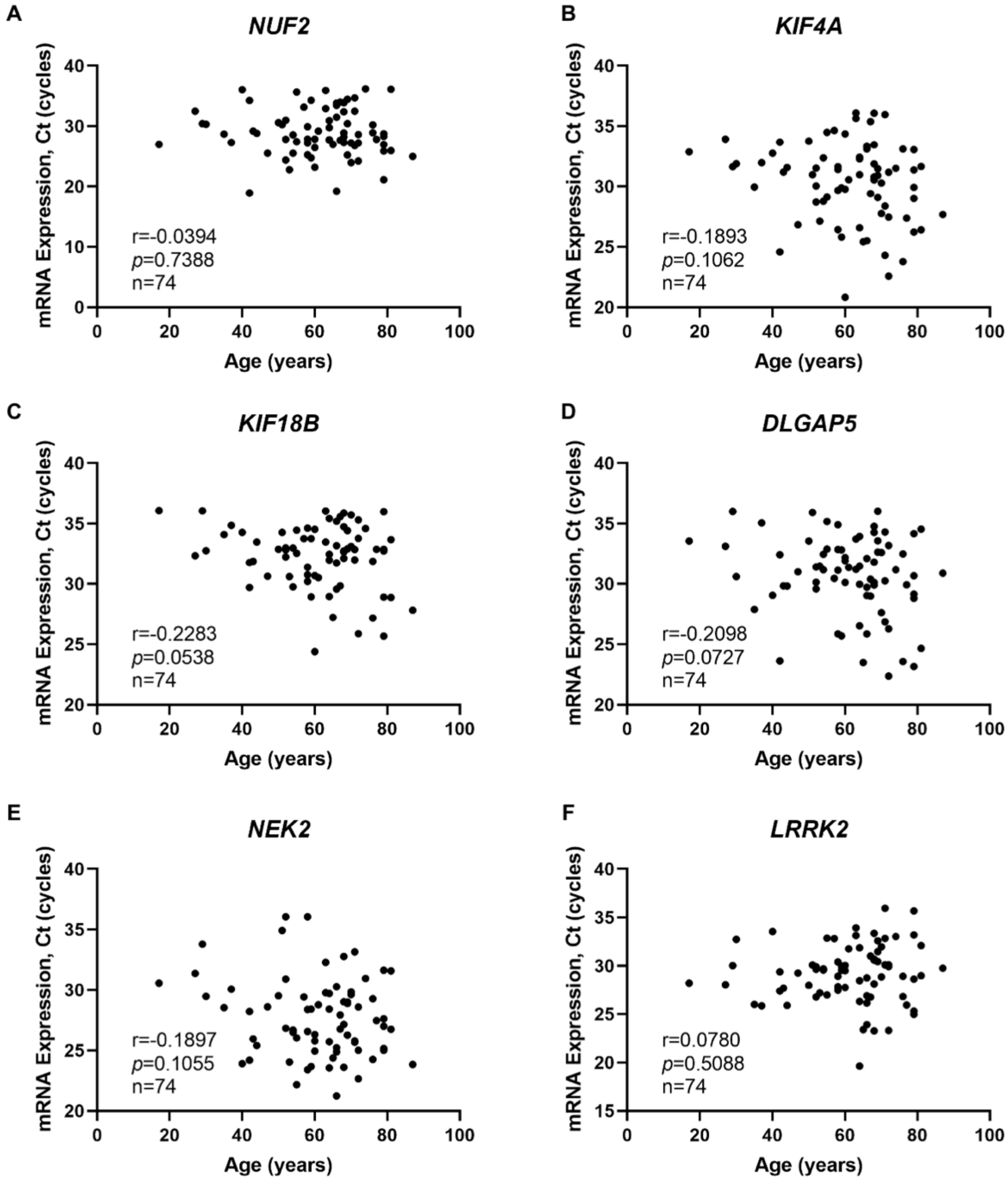

Supplementary Figure S3 Correlation analysis between age and candidate DEMG expression levels in lung cancer patients (benign=37 and malignancy=37). Pearson correlation analysis of *NUF2* (A), *KIF4A* (B), *KIF18B* (C), *DLGAP5* (D), *NEK2* (E), and *LRRK2* (F) were performed. Correlation coefficient (r), *P*-value (*p*) and number of tissue samples (n) were indicated.

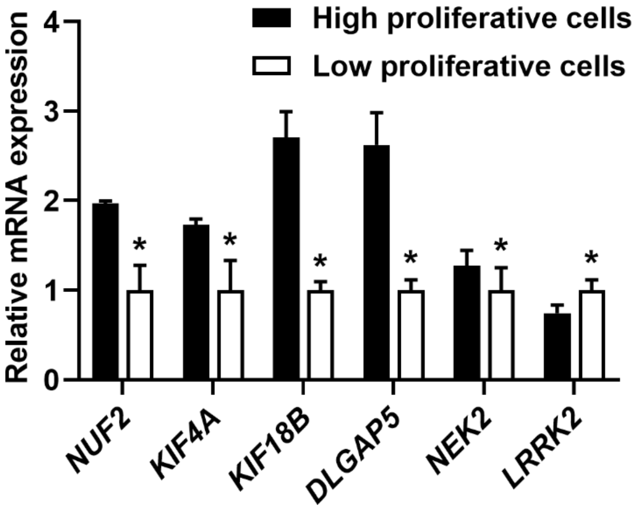

Supplementary Figure S4 The expression level of candidate DEMGs was compared between high (A549 and H460 cells) and low proliferative lung cancer cell lines (H292 and H23 cells). The mRNA expression of DEMGs were measured by qRT-PCR. * $P < 0.05$ vs. low proliferative cells (n =3).

Supplementary Table S4 Primers for qRT-PCR.

Gene	Primer sequence (5′→3′)
NUF2	F: TTTTTGCCTATCTGCCGGGT<br>R: TGTGCGGCGTTTAACTGTTG
KIF4A	F: TACTGCGGTGGAGCAAGAAG<br>R: CATCTGCGCTTGACGGAGAG
KIF18B	F: GGAGAACCGACGAAAGGTGT<br>R: AGGGTTAAACACCAGCACCC
DLGAP5	F: CCGACCTGGTCCAAGACAA<br>R: GACGTGGGCATTACAGGCT
NEK2	F: CATTGGCACAGGCTCCTAC<br>R: GAGCCATAGTCAAGTTCTTTCCA
LRRK2	F: GAAAGGCCTACTTCTGCCCA<br>R: GATGTGTAGCAACCATGCATTCA
GAPDH	F: ACATCGCTCAGACACCAT<br>R: TGTAGTTGAGGTCAATGAAGGG
